# Dosimetric Impact of Artificial Intelligence (AI)-Based Autocontouring Software, OncoStudio, in High-Risk Prostate Cancer Treatment Planning: A Three-Group Comparative Study on the Slice Ranges of Seminal Vesicles

**DOI:** 10.7759/cureus.103403

**Published:** 2026-02-11

**Authors:** Masumi Kawaguchi, Masataka Hoshina, Shinji Sugahara, Kousuke Fujioka, Masato Takanashi, Masaya Noguchi, Ayaka Oosato, Kouichi Masuda, Yoshiaki Katada, Kazuhiro Saito

**Affiliations:** 1 Department of Radiation Oncology, Tokyo Medical University Hospital, Tokyo, JPN; 2 Department of Radiation Oncology, Tokyo Medical University Ibaraki Medical Center, Ibaraki, JPN; 3 Department of Radiation, Tokyo Medical University, Tokyo, JPN; 4 Department of Radiology and Radiation Oncology, Tokyo Medical University Ibaraki Medical Center, Ibaraki, JPN; 5 Department of Radiology, Tokyo Medical University Ibaraki Medical Center, Ibaraki, JPN; 6 Department of Radiology, Tokyo Medical University, Tokyo, JPN

**Keywords:** artificial intelligence, autocontouring, deep learning, dose-volume histogram, effect size, high-risk prostate cancer, intensity-modulated radiation therapy, radiation treatment planning, slice ranges, volumetric modulated arc therapy

## Abstract

Objective: This pilot study investigated the dosimetric impact of artificial intelligence (AI)-generated seminal vesicle (SV) autocontouring (AC) compared with manual contouring (MC) in high-risk prostate cancer volumetric-modulated arc therapy planning.

Methods: We retrospectively analyzed treatment plans for 15 patients with high-risk prostate cancer who received 76 Gy in 38 fractions in intensity-modulated radiation therapy. Three plans were created for each patient: MC, AC with slice-adjustment to a clinical standard (2 cm from the prostate base), and unadjusted AC. Subsequently, three groups were made for each contouring method: the adjusted AC group, the unadjusted AC group, and the MC group, each including 15 plans. Primary endpoints were dose coverage of the planning target volume (PTV) and clinical target volume (CTV), evaluated by Dmax, Dmin, Dmean, D95, D98, D99, and V95%. Statistical analysis was performed using Dunnett's test, and multiple comparisons were made by selecting the MC as the control group.

Results: CTV Dmean showed a tendency to be lower in the unadjusted AC group compared with the MC group, but did not reach statistical significance (difference: -58.7 cGy, 95% confidence interval (CI): -123.0 to 5.5 cGy, p = 0.077, Hedges' g = 0.78). PTV Dmean also showed a decreasing tendency in the unadjusted AC group, but similarly did not reach statistical significance (difference: -92.8 cGy, 95% CI: -193.6 to 8.0 cGy, p = 0.075, g = 0.67). In the adjusted AC group, there was a slight tendency for a decrease in CTV Dmean, but this did not reach statistical significance (difference: -41.3 cGy, p = 0.254, g = 0.63). Effect size assessment revealed a moderate effect in both the unadjusted and the adjusted AC groups. No statistically significant differences were observed among the three groups in any dose volume histogram parameters.

Conclusion: This preliminary study (n = 15) suggests no statistically significant dosimetric differences between AI autocontoured and manually contoured SVs, with dose differences <1% of the prescription. However, given the small sample size, validation in larger cohorts is needed before clinical implementation.

## Introduction

In radiation therapy planning, accurate contouring of targets and organs at risk (OARs) is a critical process that holds the key to treatment success. Traditionally, contouring has been performed manually by radiation oncologists or medical physicists, but this task is time-consuming and labor-intensive, and is known to be subject to inter- and intraobserver variability [1-3].

In recent years, the rapid development of artificial intelligence (AI) has led to significant advances in automated segmentation technology in medical image processing [4,5]. In particular, the development of autocontouring (AC) software based on deep learning is expected to contribute to the efficiency and standardization of radiation therapy planning [6]. However, to promote the integration of AI-based AC in clinical settings, it is essential to evaluate its effectiveness compared with conventional manual contouring (MC) in terms of treatment plan quality [7].

In radiotherapy for high-risk prostate cancer, accurate contouring of the prostate and seminal vesicles (SVs) is an important factor directly linked to treatment outcomes [8]. Regarding the contouring slice range of the SVs, the European Organization for Research and Treatment of Cancer guidelines indicate that the clinical target volume (CTV) should include 2 cm from the base of the SVs for high-risk patients [9,10].

This recommendation is supported by pathological studies [11]. However, there are cases in which the guideline recommendations do not adequately cover the actual anatomical structures, and several publications reported variations in contouring methods among institutions [9,10]. Furthermore, most currently available AC software includes the entire SVs, which may not match the guideline-recommended range. The impact of this difference in slice range on dose distributions has not been fully investigated.

OncoStudio (Oncosoft Inc., Seoul, Republic of Korea) is a commercial AI software that employs a deep learning model using convolutional neural networks, thereby enabling automatic segmentation of the prostate and surrounding organs, including the SVs. However, the contouring slice range of the SVs does not necessarily match the standard protocol widely adopted in clinical practice (2 cm from the base of the SVs). Therefore, clinical judgment is required to determine whether to use the automatic contouring results without adjustment or to adjust them to the clinical standard.

In this study, dose volume histogram (DVH) parameters were compared among three groups: MC, adjusted AC, and unadjusted AC. Subsequently, the clinical significance of the SV contouring adjustment was evaluated for high-risk prostate cancer.

## Materials and methods

Patient selection

This single-center retrospective study was approved by the institution's ethics committee (approval number: T2025-0017). Between January 2023 and March 2025, 15 patients who underwent definitive intensity-modulated radiation therapy (IMRT) for high-risk localized prostate cancer were enrolled. Eligibility criteria were as follows: histologically confirmed high-risk prostate cancer, completed definitive IMRT at 76 Gy in 38 fractions, availability of treatment-planning CT scans, and availability of diagnostic images of sufficient image quality. Exclusion criteria were: reirradiation cases, cases with imaging artifacts, and cases requiring replanning due to small bowel involvement in the Douglas pouch.

Image acquisition and contouring

All patients underwent treatment-planning CT scans in the supine position with the bladder filled. CT scans were performed with a 1-mm slice thickness. Three different contouring methods were employed on each patient.

Manual Contouring

A radiation oncologist with over 30 years of experience manually delineated the CTV and planning target volume (PTV) on a treatment planning system (TPS) according to the institution's guidelines, where the PTV was defined by extending the CTV by 3 mm toward the posterior and 5 mm in other directions. Contouring of the SVs was performed within 2 cm of the prostate base.

Adjusted AC

The slice range of the AC results obtained using OncoStudio (version 2.0) was adjusted to match the actual clinical standard (2 cm from the base of the prostate). Specifically, the upper and lower ends of the slice level were aligned with those of the MC group, and the contours outside this slice range were manually removed. Although manual adjustment time was not quantitatively measured in this study, clinical observation suggests it involved limited modifications requiring approximately several minutes.

Unadjusted AC

The results of automatic contouring by OncoStudio were used without adjustment. In this group, the entire SV tended to be included. AI AC was completed in <30 seconds, compared with >20 minutes for complete MC.

Treatment planning and evaluation index

The treatment plan was created according to the following procedure: The results of the autocontoured Digital Imaging and Communications in Medicine Radiation Therapy structure data using OncoStudio were loaded into the Monaco TPS version 6.2 (Elekta AB, Stockholm, Sweden) via a USB memory, and then transferred to the Pinnacle3 TPS (Philips, Amsterdam, The Netherlands), which was actually used in clinical practice.

After adjusting the slice positions for the AC structure sets according to the procedure described above, the same dose optimization algorithm, SmartArc, was applied to the adjusted and unadjusted contour sets to generate two volumetric modulated arc therapy (VMAT) plans with a prescription dose of 76 Gy in 38 fractions. The following dose constraints were also applied: PTV maximum dose 75 and 77 Gy each with a weight of 100%, rectum with V70Gy < 5%, V60Gy < 10%, V50Gy < 20%, V40Gy < 30%; and bladder with V70Gy < 10%, V60Gy < 20%, V40Gy < 40%; femoral head with V25Gy = 0%, where these OAR constraints were based on the Quantitative Analysis of Normal Tissue Effects in the Clinic and Radiation Therapy Oncology Group guidelines at our institution [12,13]. Importantly, we maintained all dose constraints employed in the original treatment plan in the MC group, while adjusting only the contouring data (slice range) to optimize the dose distribution. This method allowed for a pure comparison of the contouring methods among the three groups.

For the evaluation, the following target dose coverages were compared as primary endpoints: Dmax, Dmin, Dmean, D99, D98, D95, and V95% (72.2 Gy) for PTV; Dmax, Dmin, Dmean for CTV; where V95% (72.2 Gy) shows the PTV volume coverage at 95% of the prescribed dose of 76 Gy, which is 72.2 Gy. These DVH parameters were extracted directly from the Pinnacle3 TPS and used for statistical analysis.

Statistical analysis

JMP Pro 16.0 (JMP Statistical Discovery LLC, Cary, NC) was used for statistical analysis. The MC group was designated as the control group, and Dunnett’s test [14] was performed to compare it with the adjusted and the unadjusted AC groups. Multiple comparison correction was performed automatically using the Dunnett method. For each comparison, the mean difference and 95% confidence interval (CI) were calculated. The effect size (Hedges' g) was calculated using Cohen's method [15]. First, we calculated the pooled standard deviation, then Cohen's d, and finally the small-sample correction factor $J = 1 - \frac{3}{4 (n_1 + n_2) - 9}$ to obtain Hedges' g. In this study, n_1_ = n_2_ = 15, thereby leading to J = 0.973. The interpretation for the effect size follows Cohen [15]: g < 0.2 was considered a negligible effect, 0.2 ≤ g < 0.5 a small effect, 0.5 ≤ g < 0.8 a medium effect, and g ≥ 0.8 a large effect.

Our criteria for clinically acceptable differences were set as follows: 1) PTV/CTV dose parameters: differences within ±5%, and 2) PTV V95%: differences within ±2%. In all statistical analyses, p < 0.05 was considered statistically significant. Because this study was exploratory, not only statistical significance but also effect size and clinical acceptability were comprehensively evaluated and emphasized in interpreting the results.

## Results

Patient characteristics

The patient characteristics of the 15 patients are shown in Table 1. The median age was 79 years (range: 61-86 years), and the clinical stage was T2a-T3b. The median prostate-specific antigen level was 8.34 ng/mL (range: 3.63-70.88 ng/mL). The Gleason score ranged from 3 + 4 to 5 + 5, with 4 + 4 being the most common in seven patients (46.7%), followed by 3 + 4 in three patients (20.0%), 4 + 3 in two patients (13.3%), 4 + 5 in two patients (13.3%), and 5 + 5 in one patient (6.7%). TNM classification showed T2a in eight patients (53.3%), T2c in five patients (33.3%), T3a in one patient (6.7%), and T3b in one patient (6.7%). All patients were diagnosed with high-risk prostate cancer and completed IMRT at 76 Gy in 38 fractions.

**Table 1:** Patient characteristics PSA: prostate-specific antigen

Item	Value
Median age (range)	79 years old (61-86)
Median PSA (range)	8.34 ng/mL (3.63-70.88)
Gleason score	
3 + 4	3 cases (20.0%)
4 + 3	2 cases (13.3%)
4 + 4	7 cases (46.7%)
4 + 5	2 cases (13.3%)
5 + 5	1 case (6.7%)
TNM classification	
T2a	8 cases (53.3%)
T2c	5 cases (33.3%)
T3a	1 case (6.7%)
T3b	1 case (6.7%)

Comparison of DVH parameters for target volumes

CTV Dose Parameters

The unadjusted AC group showed a tendency toward a lower CTV Dmean than the MC group, but this did not reach statistical significance (difference: -58.7 cGy; 95% CI: -123.0 to 5.5 cGy; p = 0.077). A tendency for a decrease was also observed in the adjusted AC group, but this similarly did not reach statistical significance (difference: -41.3 cGy, 95% CI: -105.5 to 23.0 cGy, p = 0.254).

PTV Dose Parameters

PTV Dmean showed a tendency to be lower in the unadjusted AC group than in the MC group, but this did not reach statistical significance (difference: -92.8 cGy, 95% CI: -193.6 to 8.0 cGy, p = 0.075). No significant differences were observed in the adjusted AC group (difference: -64.3 cGy, p = 0.258).

Other Parameters

No statistically significant differences were observed among the three groups in CTV’s Dmax and Dmin, and PTV’s Dmax, Dmin, D95, D98, D99, and V95%.

Table 2 shows a detailed comparison of DVH parameters among the three groups with Dunnett's test results. All p values were greater than 0.05, indicating no statistically significant differences among the groups.

**Table 2:** Comparison of major DVH parameters among the three groups Values are presented as mean ± SD. Dunnett's test was used for multiple comparisons. No statistically significant differences were observed (all p > 0.05) MC: manual contouring; SD: standard deviation; AC: autocontouring; CI: confidence interval; PTV: planning target volume; CTV: clinical target volume; DVH: dose volume histogram

Parameters	MC (mean ± SD)	Adjusted AC (mean ± SD)	Unadjusted AC (mean ± SD)	Adjusted AC vs. MC difference (95% CI)	Adjusted AC vs. MC p value	Unadjusted AC vs. MC difference (95% CI)	Unadjusted AC vs. MC p value
PTV Dmax (cGy)	7,825.3 ± 116.5	7,818.7 ± 115.0	7,836.1 ± 127.3	-6.6 (-106.7 to 93.4)	0.983	10.7 (-89.3 to 110.8)	0.957
PTV Dmin (cGy)	5,443.2 ± 1,052.1	5,245.3 ± 1,050.9	5,456.2 ± 834.2	-197.9 (-1020.6 to 624.8)	0.805	13.0 (-809.7 to 835.7)	0.999
PTV Dmean (cGy)	7,388.2 ± 86.0	7,323.9 ± 112.3	7,295.4 ± 153.7	-64.3 (-165.1 to 36.5)	0.258	-92.8 (-193.6 to 8.0)	0.074
PTV D99 (cGy)	6,684.2 ± 393.8	6,459.3 ± 549.1	6,615.4 ± 379.3	-224.9 (-598.8 to 149.0)	0.295	-68.8 (-442.7 to 305.1)	0.880
PTV D98 (cGy)	6,845.1 ± 331.7	6,645.3 ± 417.4	6,739.6 ± 352.5	-199.8 (-508.2 to 108.6)	0.248	-105.4 (-413.8 to 203.0)	0.653
PTV D95 (cGy)	7,050.4 ± 253.0	6,889.2 ± 320.5	6,924.5 ± 316.2	-161.2 (-410.4 to 87.9)	0.249	-125.9 (-375.1 to 123.3)	0.412
PTV V95% (%)	13.4 ± 13.0	20.7 ± 17.2	22.8 ± 20.4	7.27 (-7.06 to 21.59)	0.409	9.40 (-4.93 to 23.73)	0.240
CTV Dmax (cGy)	7,802.5 ± 116.9	7,795.1 ± 130.6	7,821.2 ± 128.2	-7.4 (-107.5 to 92.7)	0.982	18.7 (-81.4 to 118.8)	0.895
CTV Dmin (cGy)	6,857.1 ± 485.3	6,792.1 ± 436.4	6,843.4 ± 415.7	-64.9 (-438.3 to 308.4)	0.892	-13.7 (-387.1 to 359.7)	0.995
CTV Dmean (cGy)	7,457.1 ± 53.8	7,415.8 ± 72.3	7,398.3 ± 98.1	-41.3 (-105.5 to 23.0)	0.254	-58.7 (-123.0 to 5.5)	0.077

The absence of statistical significance should not be interpreted as proof of equivalence, given the limited statistical power of this small sample size (n = 15).

Effect size assessment

Hedges' g, which is a small-sample-corrected Cohen's d with a correction coefficient J = 0.973, was calculated as the effect size for the results corrected for multiple comparisons using Dunnett's test (Table 3). For CTV Dmean, g = 0.78 (medium effect) was observed for the comparison of the unadjusted AC group vs. MC group, and g = 0.63 (medium effect) was observed for the comparison of the adjusted AC group vs. MC group. For PTV Dmean, g = 0.67 (medium effect) was observed for the unadjusted AC group vs. the MC group, and g = 0.53 (medium effect) was observed for the adjusted AC group vs. the MC group. Although no statistically significant differences were observed in any comparison (all p > 0.05), moderate effect sizes were observed, suggesting clinically meaningful trends among the three groups. However, the sample size of this study was limited (n = 15), and there is uncertainty in the effect size estimates, so confirmation in larger validation studies (n ≥ 50) is needed.

**Table 3:** Effect size (Hedges' g) for CTV Dmean and PTV Dmean between MC and adjusted or unadjusted ACs Effect sizes were calculated using Hedges' g (small-sample-corrected Cohen's d, correction coefficient J = 0.973) for comparisons corrected with Dunnett's test. Effect size interpretation: small (g = 0.2), medium (g = 0.5), and large (g = 0.8). No statistically significant differences were observed in any comparison (all p > 0.05) CTV: clinical target volume; MC: manual contouring; AC: autocontouring; PTV: planning target volume

Parameters	p value	Hedges' g	Effect size
CTV Dmean (MC vs. adjusted AC)	0.254	0.63	Moderate
CTV Dmean (MC vs. unadjusted AC)	0.077	0.78	Moderate
PTV Dmean (MC vs. adjusted AC)	0.258	0.53	Moderate
PTV Dmean (MC vs. unadjusted AC)	0.075	0.67	Moderate

**Figure 1:**
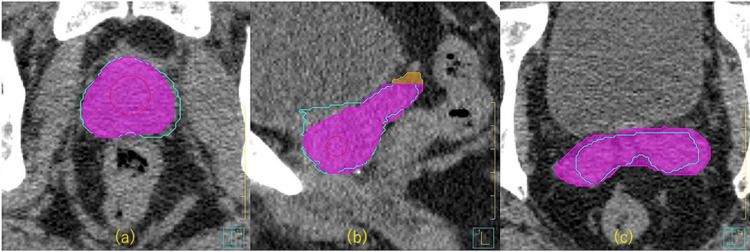
Comparison of CTV contours for MC in light blue, adjusted AC in purple, and unadjusted AC in orange, in a representative case (a) Axial view at the level of the prostate showing CTV contours: MC (light blue), adjusted AC (purple), and unadjusted AC (orange). (b) Sagittal view showing the seminal vesicles. The orange region indicates the portion of the seminal vesicles that was included by unadjusted AC but excluded in the adjusted AC group. (c) Axial view at the level of the seminal vesicles, demonstrating the extended CTV in the unadjusted AC group that includes the entire seminal vesicles. MC: light blue, adjusted AC: purple, unadjusted AC: orange CTV: clinical target volume; MC: manual contouring; AC: autocontouring

Automatic contouring by OncoStudio properly recognized anatomical boundaries and showed high agreement with MC (Figure 1).

**Figure 2:**
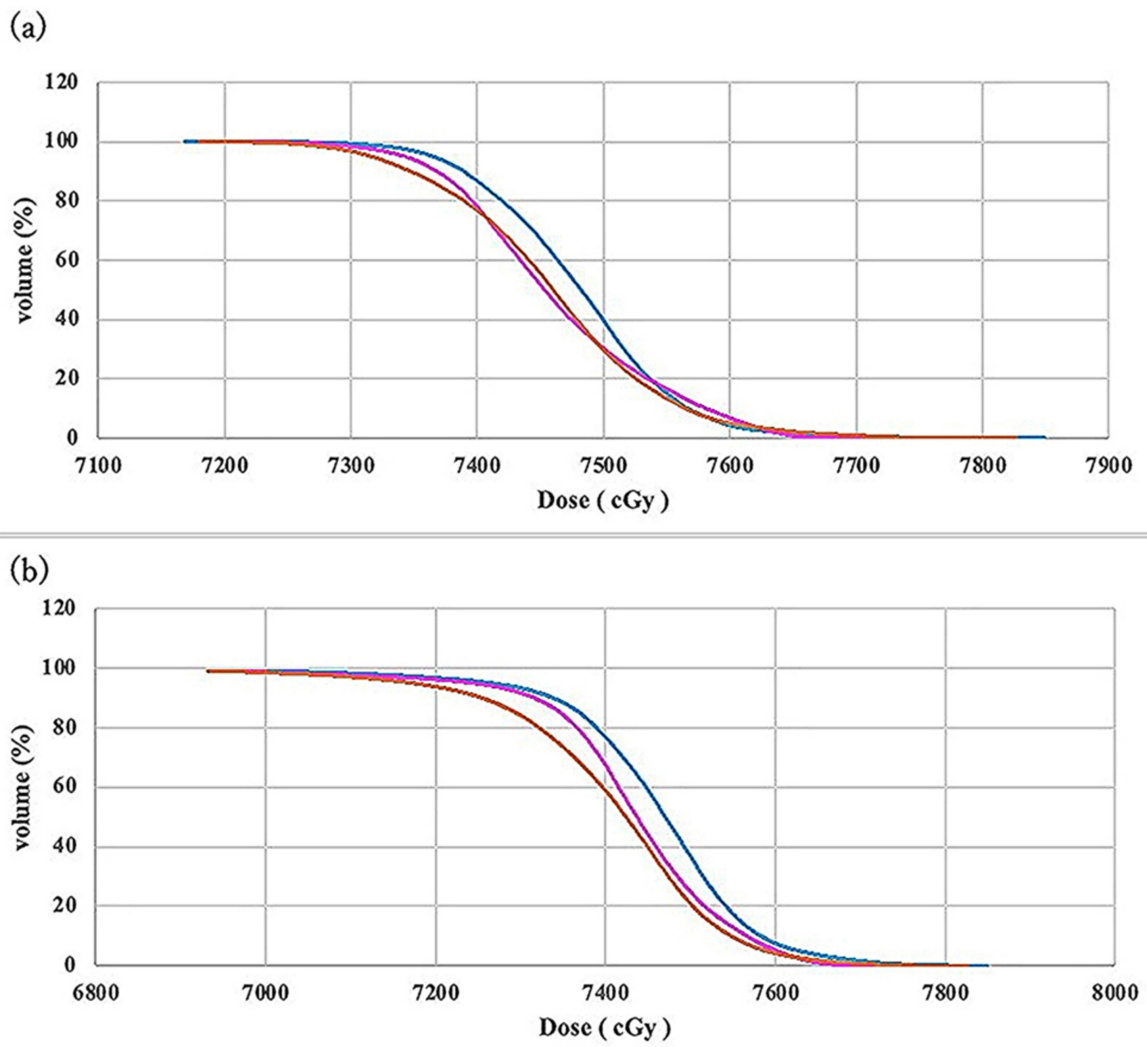
Comparison of DVH curves for (a) CTV and (b) PTV among MC (light blue), adjusted AC (purple), and unadjusted AC (orange) Notable separation among the groups was observed between 7,300 and 7,600 cGy for CTV, and 7,200 and 7,600 cGy for PTV DVH: dose volume histogram; CTV: clinical target volume; PTV: planning target volume; MC: manual contouring; AC: autocontouring

The DVH analysis revealed consistent dose patterns across all 15 cases, with the MC group demonstrating slightly higher doses than both AC groups (Figure 2).

Box plot analysis of target parameters

**Figure 3:**
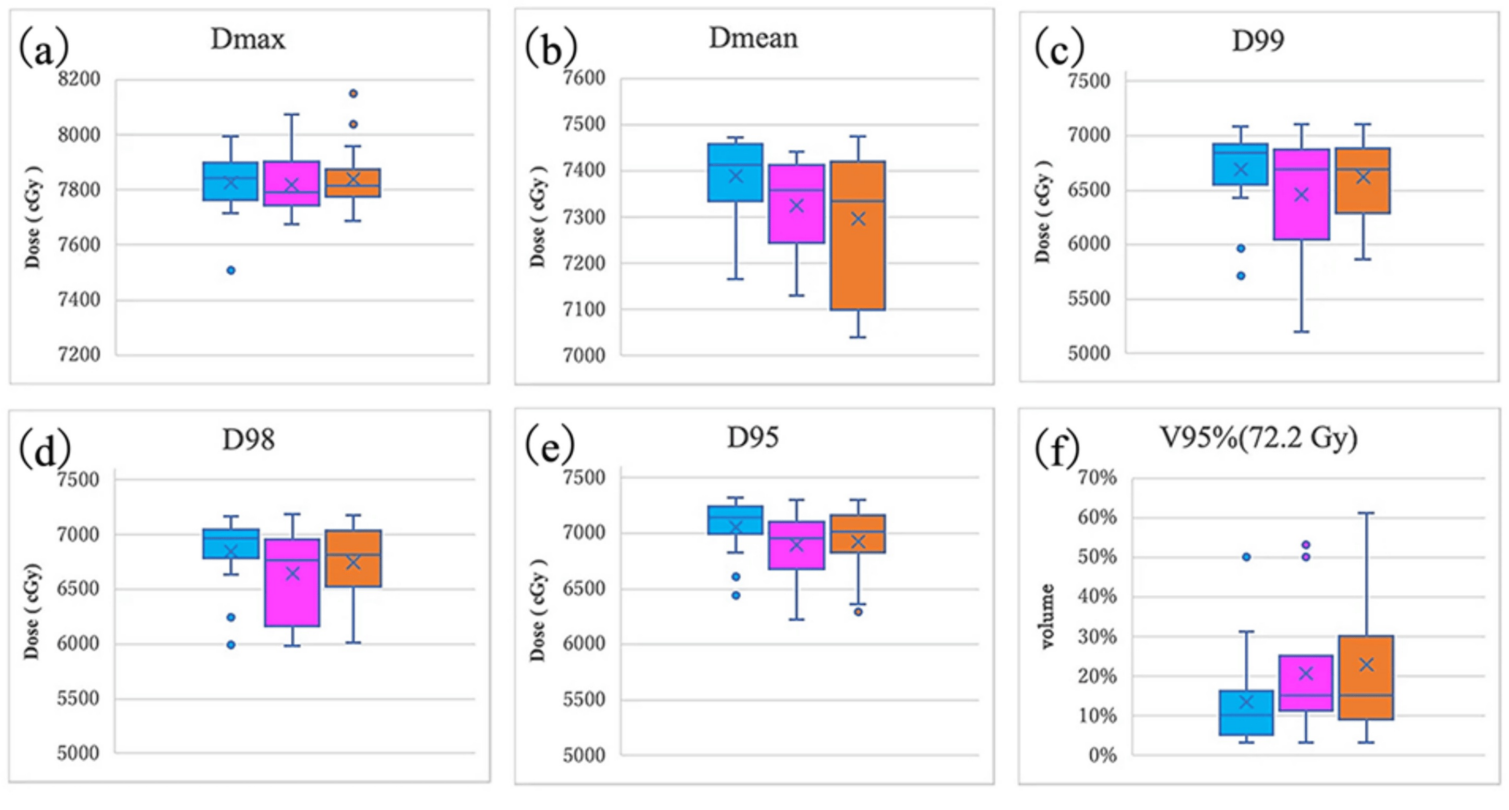
Comparison of box plots of PTV DVH parameters for MC, adjusted AC, and unadjusted AC (a) Dmax (maximum dose), (b) Dmean (mean dose), (c) D99 (dose received by 99% of the volume), (d) D98 (dose received by 98% of the volume), (e) D95 (dose received by 95% of the volume), and (f) V95% (percentage of volume receiving ≥95% of the prescribed dose, 72.2 Gy). MC: light blue, adjusted AC: purple, unadjusted AC: orange. The horizontal line indicates the median, the × indicates the mean, and dots represent outliers. No statistically significant differences were observed among the three groups (Dunnett's test, p > 0.05) DVH: dose volume histogram; PTV: planning target volume; MC: manual contouring; AC: autocontouring

**Figure 4:**
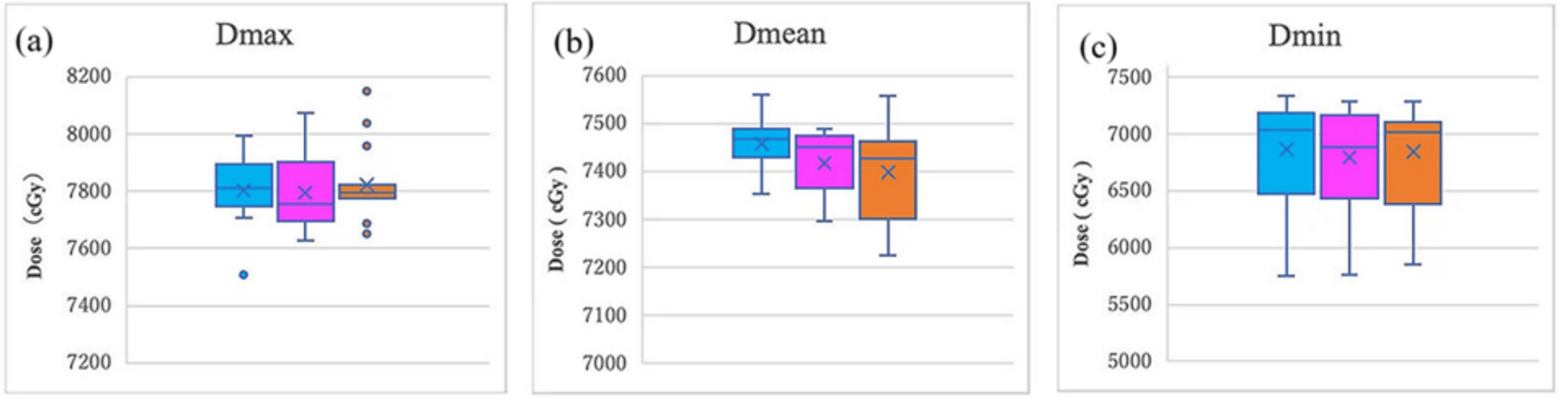
Comparisons of box plots of CTV DVH parameters for MC, adjusted AC, and unadjusted AC (a) Dmax (maximum dose), (b) Dmean (mean dose), and (c) Dmin (minimum dose). MC: light blue, adjusted AC: purple, unadjusted AC: orange. The horizontal line indicates the median, the × indicates the mean, and dots represent outliers. No statistically significant differences were observed among the three groups (Dunnett's test, p > 0.05) DVH: dose volume histogram; CTV: clinical target volume; MC: manual contouring; AC: autocontouring

To evaluate the distribution characteristics of each DVH parameter in more detail, visual analysis using box plots was performed (Figures 3, 4). Box plots were used to visually grasp the distribution of data, where the center line of the box indicates the median, and the upper and lower ends of the box indicate the third quartile (75th percentile) and the first quartile (25th percentile), respectively. The whiskers indicate the minimum and maximum values, and outliers are displayed as points. MC is shown in light blue, adjusted AC in purple, and unadjusted AC in orange.

The box plots revealed slight differences in the distribution patterns of each group. A significant decrease in the minimum value was observed in the adjusted AC group at D99 (approximately 5,000 cGy). Although a slight decrease in the minimum value was observed in the adjusted AC and unadjusted AC groups compared with the MC group at D95 (approximately 6,400 cGy for the MC group and approximately 6,200 cGy for the adjusted AC and unadjusted AC groups), the difference was only approximately 200 cGy, which was not considered clinically significant. No clear differences in the minimum value were observed among the three groups at D98. A tendency for the minimum value and overall distribution to decrease in the unadjusted AC group was observed for the CTV Dmean and PTV Dmean.

These findings suggest that the SV contouring range may affect dose distribution at the individual case level in some cases. However, the overall median and interquartile range were similar among the three groups. Although no statistically significant differences were observed in the mean PTV V95% among the three groups (p > 0.05), a comparison of medians using boxplots revealed a tendency for the adjusted AC and unadjusted AC groups (median approximately 20%) to have slightly higher values than the MC group (median approximately 10%). However, the adjusted AC group contained significant outliers (50%-60%) and exhibited asymmetric data distribution, resulting in different trends between the mean and median values.

Clinically, all groups were within the established tolerance range (±2%), suggesting that the impact of differences in SV contouring methods on volume coverage at 95% of the prescribed dose is limited. Distribution analysis using boxplots visualizes patterns of variation at the individual case level that cannot be detected by statistical testing. The adjusted AC group showed distribution characteristics closer to those of the MC group, supporting the effectiveness of SV area adjustment. Figure 3 shows the comparisons of box plots of PTV DVH parameters for MC, adjusted AC, and unadjusted AC. Figure 4 compares the box plots of CTV DVH parameters for MC, adjusted AC, and unadjusted AC.

Assessment of clinical tolerance

Evaluation based on established clinical acceptability criteria yielded the following results.

Adjusted AC Group

The difference in CTV Dmean (-41.3 cGy) was equivalent to 0.5% of the prescribed dose and was considered within the clinically acceptable range (±5%). The differences in PTV-related parameters were also within the acceptable range.

Unadjusted AC Group

The difference in CTV Dmean (-58.7 cGy) was equivalent to 0.8% of the prescribed dose, which is within the clinically acceptable range (±5%). Although no statistically significant difference was observed, a decreasing tendency was noted, suggesting that attention may be warranted in clinical use. In the V95% parameter, the difference between both groups and the MC group was within 2%, meeting the established acceptable range criteria.

## Discussion

In this study, we performed a clinical evaluation of SV contouring adjustment using AI-based automated contouring software (OncoStudio) in radiotherapy planning for high-risk prostate cancer. The main finding was that no statistically significant differences were observed among the three groups, indicating that AC can achieve dose distributions comparable to those of MC.

Effect of SV contouring range

The tendency for decreased CTV Dmean and PTV Dmean observed in the unadjusted AC group (although not statistically significant) suggests that including the entire SVs may result in a slightly larger target volume. OncoStudio tends to automatically include the entire SVs; however, the results of this study demonstrated that the impact of this difference on dose distribution was within clinically acceptable ranges.

Usefulness of adjusted AC

In the adjusted AC group, a dose distribution closer to that in the MC group was obtained. Although no statistically significant difference was observed, the effect sizes (g = 0.53-0.63) suggest that SV slice range adjustment may contribute to improving dose distribution agreement with MC.

Clinical equivalence among the three groups

An important finding of this study was that no statistically significant differences were observed among the three groups in Dunnett's test multiple comparison analysis. This suggests that AC by OncoStudio (both adjusted and unadjusted) can produce results within clinically acceptable ranges compared to MC performed by a radiation oncologist with over 30 years of experience, although the moderate effect sizes observed suggest that verification in larger studies is warranted. This result supports the clinical utility of AI-based AC.

Comparison with previous studies

Our findings are consistent with previous studies that evaluated the dosimetric impact of deep learning-based AC in prostate cancer radiotherapy. Zabel et al. compared manual, deep learning, and atlas-based contouring workflows in 15 prostate cancer patients and reported no differences in clinically relevant dose-volume metrics between workflows [16]. In their study, the mean duration of initial contour generation was significantly shorter with deep learning (1.4 minutes) than with MC (10.9 minutes), while maintaining comparable dosimetric accuracy. Similarly, Kawula et al. investigated the dosimetric impact of 3D U-Net-based autosegmentation on radiation therapy treatment planning for prostate cancer and demonstrated that DVH parameters showed agreement between manual and automatic segmentations, with no excessive dose increase to OARs and no substantial under- or overdosage of the target [17]. These findings, together with our results, support the clinical feasibility of AI-based AC for prostate cancer radiotherapy planning.

Notably, Kawula et al. emphasized the importance of combining geometric and dosimetric analyses, as no strong statistically significant correlation between these metrics was observed [17]. This suggests that geometric accuracy alone may not fully predict dosimetric outcomes, highlighting the need for comprehensive evaluation approaches such as those employed in the present study. To the best of our knowledge, no previous studies have compared DVH parameters among MC, AC with the entire SV contoured, and AC with only the proximal 2-cm portion of the SV from the base of the prostate contoured. Our study focused specifically on this comparison, providing novel insights into the impact of SV slice range adjustment on dosimetric outcomes.

The moderate effect sizes observed in our study (Hedges' g = 0.53-0.78), despite the lack of statistical significance, are noteworthy. This discrepancy between statistical significance and effect size may reflect the limited statistical power due to the small sample size (n = 15), which is comparable to that used by Zabel et al. [16]. Verification in larger cohorts is warranted, as also recommended by previous studies [16,17].

Advantages of MC

The MC group showed the best dose distribution in the DVH analysis. The following are possible reasons. First, in the MC group, precise contouring by an experienced radiation oncologist ensured that only the clinically necessary and sufficient slice range was set as the target volume. In the unadjusted AC group, the target volume was oversized by including the entire SVs, and the unnecessarily enlarged target volume may have made it difficult to optimize dose conformity to the target while satisfying dose constraints to the OARs. In the adjusted AC group, subtle differences in range setting during manual adjustment may have led to suboptimal dose conformity.

Clinical implications

The results of this study provide important insights into the clinical implementation of automated contouring technology. Rather than simply using the results of automated contouring software, appropriate adjustments based on the institution's standard guidelines are necessary. The combination of automated technology and clinical judgment is crucial, especially for anatomically complex structures such as the SVs.

Statistical considerations

Setting MC as the control group allowed for efficient evaluation of the clinical value of the two AC methods. Multiple comparison correction was appropriately performed using the Dunnett method, and the use of effect size, along with statistical significance, enabled robust evaluation that does not rely solely on p values.

Clinical significance of effect size

In this study, we evaluated effect size (Hedges' g) in addition to statistical significance. Although no statistically significant differences were observed in any comparison (all p > 0.05), moderate effect sizes (g = 0.53-0.78) were observed. This may reflect the limited statistical power due to the small sample size (n = 15), and verification in larger studies is needed. Importantly, all observed differences were within clinically acceptable ranges (less than 1% of the prescribed dose).

Limitations

This study has several important limitations that must be acknowledged. First, the most notable weakness is the small sample size (n = 15). Although we mitigated small-sample bias using Hedges' g, the risk of Type II error (false negative) remains. The moderate effect sizes (g = 0.53-0.78) observed in unadjusted AI contours suggest a trend toward dose reduction, but our study lacks statistical power to definitively confirm or exclude subtle dosimetric differences. Therefore, this study should be positioned as a preliminary validation rather than a definitive equivalence trial. Second, the time efficiency of the adjusted workflow was not quantitatively assessed. While AI AC clearly completes faster than MC (<30 seconds vs. >20 minutes), manual adjustment of SV range in the Adjusted AC group involved limited modifications that, based on clinical experience, are estimated to require approximately several minutes. Thus, overall time efficiency (30 seconds + several minutes vs. manual >20 minutes) is likely maintained. However, without precise measurement data, we cannot confirm this assumption. Future studies should prospectively measure the specific time required. Third, manual contours (MC group) and AI contour adjustments were performed by a single radiation oncologist, which may reflect individual contouring style rather than true ground truth. Ideally, consensus contours from multiple experts should be used as the gold standard. Fourth, this study was not designed as a statistical equivalence trial and was not based on predefined equivalence margins or power calculations. Therefore, the results should be interpreted as "suggesting clinical acceptability" rather than "proving equivalence."

Future prospects

Future research will include validation through larger multicenter prospective studies and the development and implementation of an automated slice-range adjustment algorithm. Additionally, comparisons with other automatic contouring software are an important research topic.

## Conclusions

This pilot study (n = 15) found no statistically significant dosimetric differences between AI autocontoured and manually contoured SVs in high-risk prostate cancer VMAT planning, with dose differences <1% of the prescription. Despite geometric discrepancies between AI-generated contours and clinical guidelines, the observed dosimetric equivalence suggests that extended cranial SV coverage did not result in clinically meaningful dose escalation to adjacent OARs. While AI AC is substantially faster than manual methods (<30 seconds vs. >20 minutes), these preliminary findings require validation in larger prospective cohorts before routine clinical implementation. Future studies should include formal equivalence testing, time-efficiency analysis, and multiobserver validation.
